# Climate Change Impacts Indoor Environment

**DOI:** 10.1289/ehp.119-a382

**Published:** 2011-09-01

**Authors:** Carol Potera

**Affiliations:** Carol Potera, based in Montana, has written for *EHP* since 1996. She also writes for *Microbe*, *Genetic Engineering News*, and the *American Journal of Nursing*.

For many years investigators have been aware of potential links between climate change and outdoor air quality.^1^ Far fewer studies have focused on climate change and indoor air quality, but a new report from the Institute of Medicine (IOM) concludes that the relationship between the two warrants further attention and action.^2^

“There’s not much research at this interface, and hard evidence is needed,” says John Spengler, an atmospheric scientist at the Harvard School of Public Health, who chaired the committee that authored the report. “This report identifies indoor air quality as a priority that deserves an important place in climate change research and policy.”

The U.S. Environmental Protection Agency asked the IOM to independently investigate the issue. “Most people spend the majority of their time indoors, so it makes sense that people will experience climate change from a housing perspective,” says Patricia Butterfield, dean of the Washington State University College of Nursing, who reviewed the report. The IOM committee describes potential changes to residential and commercial buildings resulting from efforts either to mitigate or adapt to climate change.

Climate change mitigation plans seeking to reduce carbon dioxide emissions often tout the goal of reducing the amount of energy needed to maintain a comfortable indoor environment. That’s because coal combustion for electricity production is a primary source of U.S. emissions of carbon dioxide.^3^ But steps such as weatherizing buildings to make them more energy efficient could create new indoor problems or worsen existing conditions, according to the report. For example, caulking and sealing leaks in buildings may alter airflow and concentrate indoor pollutants such as tobacco smoke, radon, and chemical emissions from building materials.^4^ And trapped moisture can spur mold and bacterial growth.^5^

**Figure:**
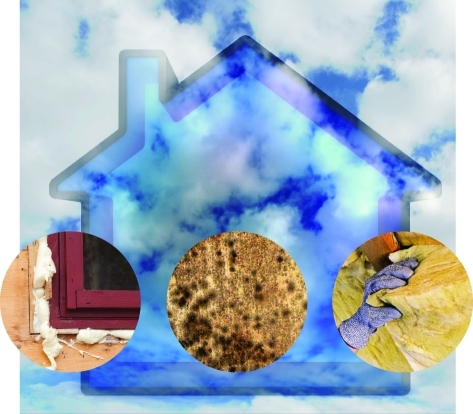
Weatherproofing must be undertaken with care to avoid unintended problems with indoor air quality. Shuttersock.com; Joseph Tart/EHP

Severe weather presents another opportunity for indoor air hazards. For instance, Butterfield says, families may face an increased likelihood of flooded basements or mold in attics related to predicted increases in extreme weather. And an increase in cases of carbon monoxide poisoning after hurricanes has been traced to the improper use of portable gasoline-powered generators, which emit high levels of carbon monoxide.^6^ When generators are used properly with good ventilation, they are not a problem. But when used improperly close to or inside homes, people end up in emergency rooms or even dead. “It’s a good example of the interplay we will experience as we adapt to climate change,” Spengler says. He adds that new weatherizing materials and techniques may be commercialized faster than their health implications can be assessed. “We will invent all sorts of things as we adapt to mitigate climate change,” he explains.

The report authors write that an “upfront investment” is needed to consider the potential consequences of housing-related adaptation actions in order to avoid problems and prevent the costs of medical care and lost productivity of building occupants. This investment might entail research that combines data from government agencies to understand how climate change affects environmental health, putting programs in place to certify products as helpful or nonhazardous, and training workers to properly install proven products. Models to ensure the safety of building materials already exist in the U.S. Green Building Council and Labs21 programs.^7^

The report urges government agencies to cooperate in investigating the problem and work toward solutions. Several agencies already collect extensive data sets, such as residential energy consumption surveys conducted by the Department of Energy^8^ and the National Hospital Discharge Survey compiled by the National Center for Health Statistics of the Centers for Disease Control and Prevention.^9^ Combining such data could help to answer questions about how climate change affects indoor health.

Identifying complex public health issues that connect global climate change and indoor air environments can lead to sound policy decisions that could save lives, Butterfield says. “The IOM report makes a connection between global climate change and weather extremes and the behavior of people as they try to adapt,” she says. “Unfortunately, many people will experience climate change as a natural disaster like a flood or hurricane. We need to connect global climate change with the indoor air environment to give thoughtful guidance to people.”

## References

[1] Penner JE (1989). Climate Change and Its Interactions with Air Chemistry: Perspectives and Research Needs. Project Summary..

[2] Committee on the Effect of Climate Change on Indoor Air Quality and Public Health, Institute of Medicine. Climate Change, the Indoor Environment, and Health. Washington, DC:The National Academies Press (2011). Available: http://tinyurl.com/4xn4rhj [accessed 8 Aug 2011].

[3] EPA. 2011 U.S. Greenhouse Gas Emissions. Greenhouse Gas Inventory Report [website]. Washington, DC:U.S. Environmental Protection Agency (updated 5 Aug 2011). Available: http://tinyurl.com/coyr8r [accessed 8 Aug 2011].

[4] Letz GA (1990). Sick building syndrome: acute illness among office workers—the role of building ventilation, airborne contaminants and work stress.. Allergy Proc.

[5] Institute of Medicine, Committee on Damp Indoor Spaces and Health. Damp Indoor Spaces and Health. Washington, DC:National Academies Press (2004). Available: http://tinyurl.com/3mmnabz [accessed 8 Aug 2011].

[6] Centers for Disease Control and Prevention (2005). Carbon monoxide poisoning after Hurricane Katrina—Alabama, Louisiana, and Mississippi, August–September 2005.. MMWR.

[7] The Environmental Protection Agency and the Department of Energy first sponsored these organizations, which now operate independently. The U.S. Green Building Council backs the green building industry through environmentally responsible materials, sustainable architecture, and public policy, and Labs21 promotes the design of low-energy laboratories.

[8] EIA. Residential Energy Consumption Survey (RECS) [website]. Washington, DC:U.S. Energy Information Administration, U.S. Department of Energy (2009). Available: http://tinyurl.com/6xpex3c [accessed 8 Aug 2011].

[9] NCHS. National Hospital Discharge Survey [website]. Hyattsville, MD:National Center for Health Statistics, U.S. Centers for Disease Control and Prevention (updated 21 Jun 2011). Available: http://tinyurl.com/3prz22o [accessed 8 Aug 2011].

